# Supplementary material for: H2A-H2B Histone Dimer Plasticity and Its Functional Implications
Source: Cells. 2022 Sep 12;11(18):2837. doi: 10.3390/cells11182837 (PMC9496766; doi:10.3390/cells11182837)
Supplement: Supplementary file 1 [file cells-11-02837-s001.zip › cells-1890073-supplementary.pdf]

SUPPLEMENTARY INFORMATION

H2A-H2B histone dimer plasticity and its functional implications

Anastasiia S. Kniazeva[1], Grigoriy A. Armeev [2] and Alexey K. Shaytan [3*]

[1] Department of Biology, Lomonosov Moscow State University, Moscow, Russia, 119234;
[2] Department of Biology, Lomonosov Moscow State University, Moscow, Russia, 119234;
[3] Department of Biology, Lomonosov Moscow State University, Moscow, Russia, 119234;
* Correspondence: shaytan_ak@mail.bio.msu.ru ; Tel.: +7(495)9395738

Table S1. List of systems used in MD simulations and achieved trajectory times. PDB ID is the identifier of the initial structure that was used as initial structure for MD simulations. The initial structures were altered by removing histone tails or DNA or modifying the conformation of DNA. The second part of the table lists the previously published [1] and analyzed in this paper MD trajectories.

System	Description	PDB ID	MD trajectory time, µs
H2A-H2B$^{tt}_{1KX5}$	H2A-H2B dimer, truncated histone tails	1KX5	3
H2A-H2B$^{tt}_{3LZ0}$	H2A-H2B dimer, truncated histone tails	3LZ0	3
H2A-H2B/DNA$^{tt}_{1KX5}$	H2A-H2B dimer with 30 bp DNA fragment, truncated histone tails	1KX5	3
H2A.Z-H2B$^{tt}_{1F66}$	H2A.Z-H2B dimer, truncated histone tails	1F66	3
NCP$^{tt}_{H2A.Z}$	Nucleosome core particle with H2A.Z histones, truncated histone tails, 601 Widom DNA sequence	1F66/3LZ0	5
NCP$^{tt}_{25\ unwrapped}$	Nucleosome core particle with canonical histones, truncated histone tails, 25 bp DNA fragment is unwrapped in initial state	3LZ0	1.8
NCP$^{tt}_{50\ unwrapped}$	Nucleosome core particle with canonical histones, truncated histone tails, 50 bp DNA fragment is unwrapped in initial state	3LZ0	1.2
Previously published MD trajectories			
NCP$_{147}$	Nucleosome core particle with canonical histones, full-length histone tails in symmetric starting positions, 147 bp quasi-palindromic α-satellite DNA in pseudo-symmetric conformation	1KX5	15
NCP$^{tt}_{147}$	Nucleosome core particle with canonical histones, truncated histone tails, 147 bp quasi-palindromic α-satellite DNA in pseudo-symmetric conformation	1KX5	10
NCP$^{tt}_{146}$	Nucleosome core particle with canonical histones, truncated histone tails, 146 bp palindromic α-satellite DNA sequence	1AOI	15
NCP$^{tt}_{145}$	Nucleosome core particle with canonical histones, truncated histone tails, 145 bp Widom 601 DNA sequence	3LZ0	8

Table S2. List of PDB codes of experimental structures with H2A and H2B histones used for analysis.

Category	PDB ID list
Dimer within free NCPs (EM and XRD)	3WTP, 5X7X, 3AV2, 5XM0, 3AV1, 5AVC, 5B0Z, 5AV6, 5XF4, 5CPJ, 3AZJ, 6T93, 5AV8, 6PWE, 4KGC, 5B24, 3W96, 5JRG, 3AZM, 6JR0, 5Y0D, 5B2I, 7KBE, 7NL0, 5XF3, 6R94, 2CV5, 5CPI, 5AV5, 5XF5, 5B1M, 5CPK, 3AZK, 3W99, 1EQZ, 3AZI, 5AV9, 3W97, 3AFA, 3AZN, 7LYA, 6JR1, 3AZL, 6KVD, 5Y0C, 7KBD, 4YM6, 5B2J, 6V2K, 5AVB, 3AZF, 3AZH, 3AZG, 3AZE, 3AYW, 3REI, 2NZD, 3MGQ, 6ESF, 4XUJ, 3MGS, 3LZ0, 1S32, 4J8V, 6WZ5, 3O62, 7OHC, 3UTA, 3B6F, 2FJ7, 5DNM, 4WU8, 1KX4, 5XM1, 3X1S, 4J8X, 3LJA, 3REL, 5CP6, 3UT9, 3REJ, 2PYO, 3REH, 3KUY, 3MGP, 6WZ9, 3LZ1, 3MNN, 3MGR, 4WU9, 2NQB, 4J8U, 3UTB, 3B6G, 1KX5, 6PX1, 3C1B, 1KX3, 5XF6, 5DNN, 3REK, 4J8W, 3KXB, 5B0Y, 5GXQ, 6T79, 5B40, 1M1A, 5B1L, 3C1C, 1M19, 5F99, 3A6N, 5OMX, 7SWY, 1M18, 1P3P, 1P3G, 1P3I, 1P3O, 1P3B, 1P3F, 5ONW, 5ONG, 1P3A, 1P3K, 1P34, 1P3M, 1P3L, 4Z5T, 7DBH, 7VBM, 5ZBX, 5AY8, 1AOI, 5Z23, 3W98, 7XD1, 6FQ5, 6R1T, 7KTQ, 6IPU, 4XZQ, 4Z66, 6JXD, 4YS3, 6IQ4, 3KWQ, 6KE9, 6LE9, 6L9H, 6KXV, 3AN2, 6SE0, 6SEG, 6O1D, 6TEM
Dimer within complexes (EM and XRD)	6IR9, 6RNY, 7D1Z, 6RYR, 6YOV, 5E5A, 6R8Z, 6NE3, 6T90, 4LD9, 7JOA, 7LYC, 5GTC, 7JO9, 5X0X, 7A08, 5Z3O, 3MVD, 6K1P, 6T9L, 6JYL, 6O96, 6NOG, 4R8P, 6PA7, 6IRO, 6NZO, 3TU4, 6NN6, 6VZ4, 6KIU, 6ZHX, 6ZHY, 6UH5, 6UGM, 6T7A, 1ZLA, 6S01, 6NQA, 6VEN, 7JZV, 6NJ9, 6X59, 7CRQ, 7CRO, 7CRR, 7CRP, 7CCQ, 6IY3, 6IY2, 7K6Q, 7K6P, 5MLU, 6Y5E, 6SE6, 6MUO, 7D20, 6BUZ, 6SEF, 6MUP
NCPs with Widom 601 sequence	7K5Y, 7K63, 7K5X, 7K60, 4QLC, 6IR9, 7D1Z, 6RYR, 7SCZ, 6T93, 6YOV, 6PWE, 6NE3, 7LYB, 7NKY, 7K61, 7KBE, 6T90, 4LD9, 7JOA, 6DZT, 7NKX, 7LYA, 7LYC, 7KBD, 7JO9, 5X0X, 7A08, 7EG6, 5Z3O, 6ESF, 7ENN, 3LZ0, 7OHA, 6WZ5, 7OHC, 7E8I, 7MBN, 3MVD, 6K1P, 6T9L, 6JYL, 6O96, 6NOG, 4R8P, 7OH9, 3UT9, 6PA7, 6WZ9, 3LZ1, 6IRO, 6NZO, 7MBM, 3TU4, 7OHB, 6PX1, 6NN6, 6VZ4, 6KIU, 6ZHX, 6ZHY, 6UH5, 6UGM, 7E8D, 6S01, 7TN2, 7SWY, 6NQA, 6VEN, 7JZV, 6NJ9, 6X59, 7CRQ, 7CRO, 7CRR, 7CRP, 7DBH, 7VBM, 7EA8, 7EA5, 7XD1, 6FQ5, 7XD0, 6R1T, 7KTQ, 6JXD, 7CCQ, 7XCR, 7XCT, 7K6Q, 7K6P, 5MLU, 6Y5E, 6SE0, 6SEG, 6SE6, 7D20, 6TEM, 6BUZ, 6E0C, 6SEF
NCPs with alpha-satellite sequence 146 bp.	3WTP, 5X7X, 3AV2, 5XM0, 3AV1, 5B0Z, 3AZJ, 5E5A, 5B24, 3W96, 5JRG, 6R8Z, 3AZM, 6JR0, 5Y0D, 5B2I, 6R94, 2CV5, 5B1M, 3AZK, 3W99, 1EQZ, 3AZI, 3W97, 3AFA, 3AZN, 6JR1, 3AZL, 6KVD, 5GTC, 5Y0C, 4YM6, 5B2J, 6V2K, 3AZF, 3AZH, 3AZG, 3AZE, 3AYW, 1S32, 3O62, 1KX4, 5XM1, 3X1S, 3REL, 3REJ, 2NQB, 3UTB, 3C1B, 1KX3, 3REK, 3KXB, 5B0Y, 5GXQ, 5B40, 1M1A, 1ZLA, 5B1L, 3C1C, 1M19, 3A6N, 1M18, 1P3P, 1P3G, 1P3I, 1P3O, 1P3B, 1P3F, 1P3A, 1P3K, 1P34, 1P3M, 1P3L, 4Z5T, 5ZBX, 5AY8, 1AOI, 5Z23, 3W98, 3KWQ, 6KXV
NCPs with alpha-satellite sequence 147 bp.	5AVC, 5AV6, 5XF4, 5AV8, 4KGC, 5XF3, 5AV5, 5XF5, 5AV9, 5AVB, 3REI, 2NZD, 3MGQ, 4XUJ, 3MGS, 4J8V, 3UTA, 3B6F, 5DNM, 4WU8, 4J8X, 3LJA, 5CP6, 2PYO, 3REH, 3KUY, 3MGP, 3MNN, 3MGR, 4WU9, 4J8U, 3B6G, 1KX5, 5XF6, 5DNN, 4J8W, 5OMX, 5ONW, 5ONG, 6IPU, 4XZQ, 4Z66, 4YS3, 6IQ4, 7U46
Free dimer (NMR and XRD)	2RVQ, 7PJ1, 6K01, 7BP2

Dimer within complexes (EM and XRD)	7QXA, 7QXS, 7QXB, 7BG9, 7TRF, 6N1Z, 7V99, 4KHA, 5BT1, 6KBB,7BP6, 6M2M, 7BP5, 7BP4, 7C7X, 5FUG, 4CAY, 4WNN

Table S3. Significance of H2A-H2B α2-α2-angle values differences between free dimer, dimer with DNA and NCP-embedded dimers in MD simulations.

	two-sample Kolmogorov-Smirnov test		T-test for the means of two independent samples	
System types	statistic	p-value	statistic	p-value
Dimer - NCP	0.36	<0.001	-8.11	<0.001
Dimer - Dimer+DNA	0.50	<0.001	-12.01	<0.001
NCP - Dimer+DNA	0.25	0.001	3.20	0.002

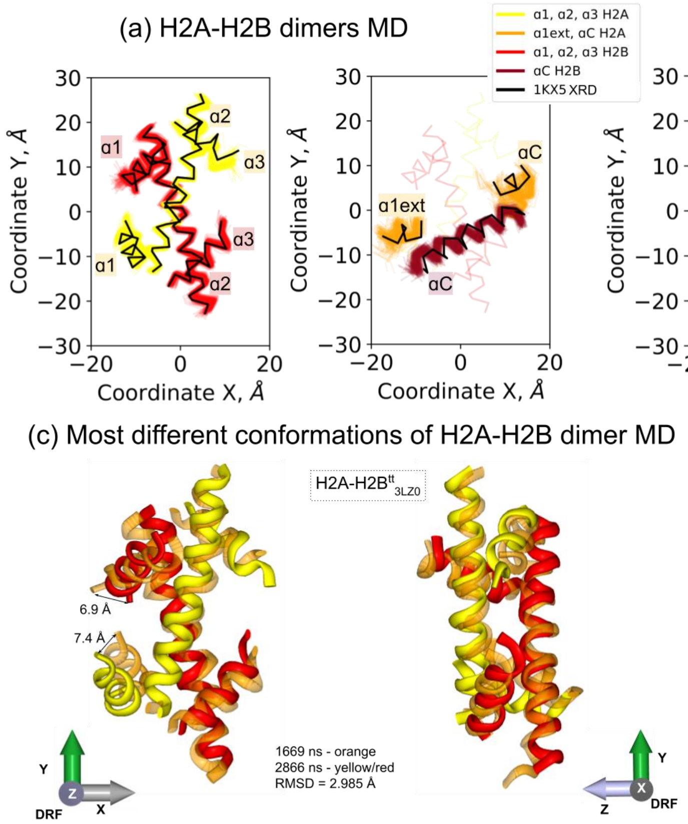

Figure S1. Plasticity of H2A-H2B dimer in MD simulations. (a,b) 2D projections of H2A and H2B histone α-helices conformations for free H2A-H2B dimers (a) and NCPs (b). **(c)** Overlay of the most different conformations of H2A-H2B globular domain in the dimer MD simulation.

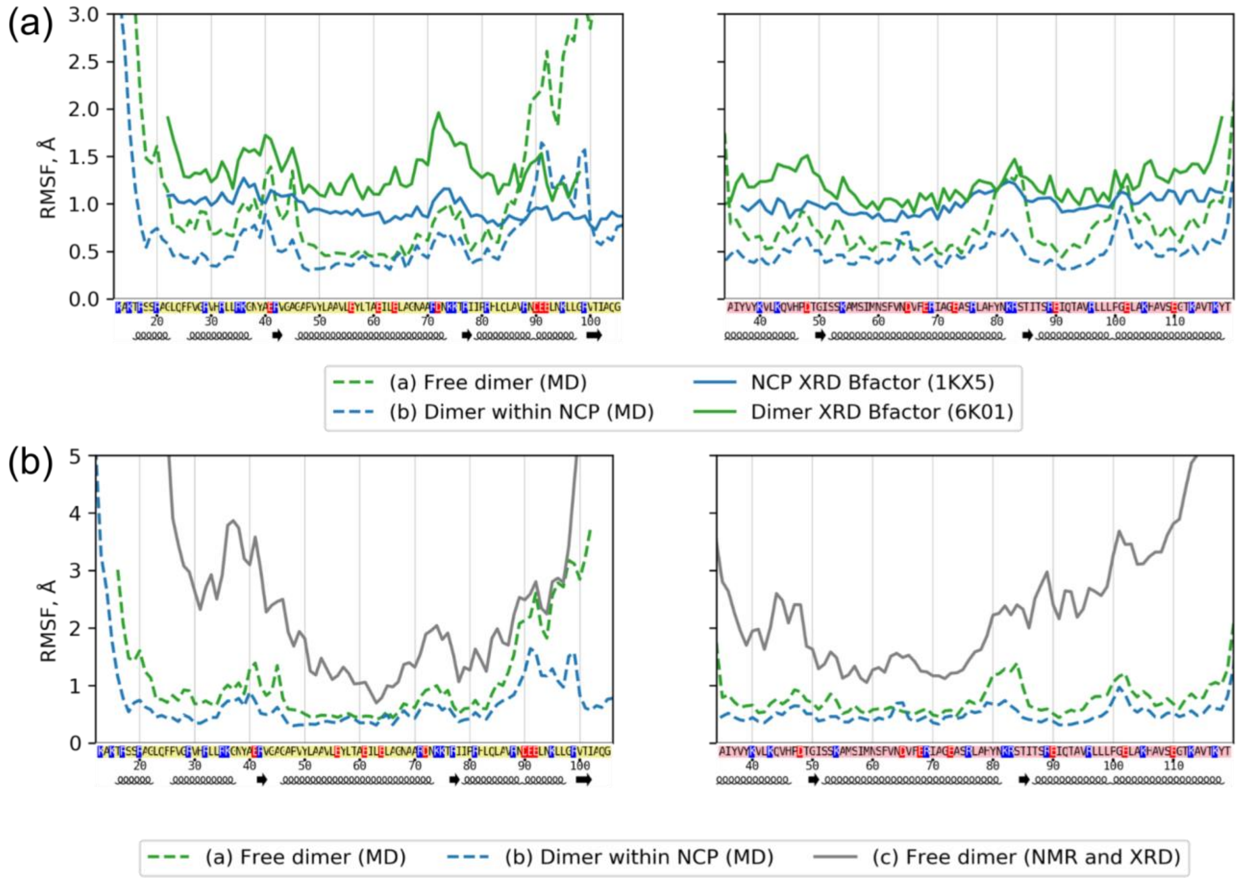

Figure S2. Cα-atoms RMSF profiles of H2A and H2B histones in MD simulations and experimental structures from Protein Data Bank. (a) Profiles of free H2A-H2B dimers and NCP-embedded dimers from MD and high resolution X-ray diffraction data. RMSF calculations for X-ray structures used reported B-factors (see Methods). (b) RMSF profiles of the reported NMR structures ensembles of free H2A-H2B dimers.

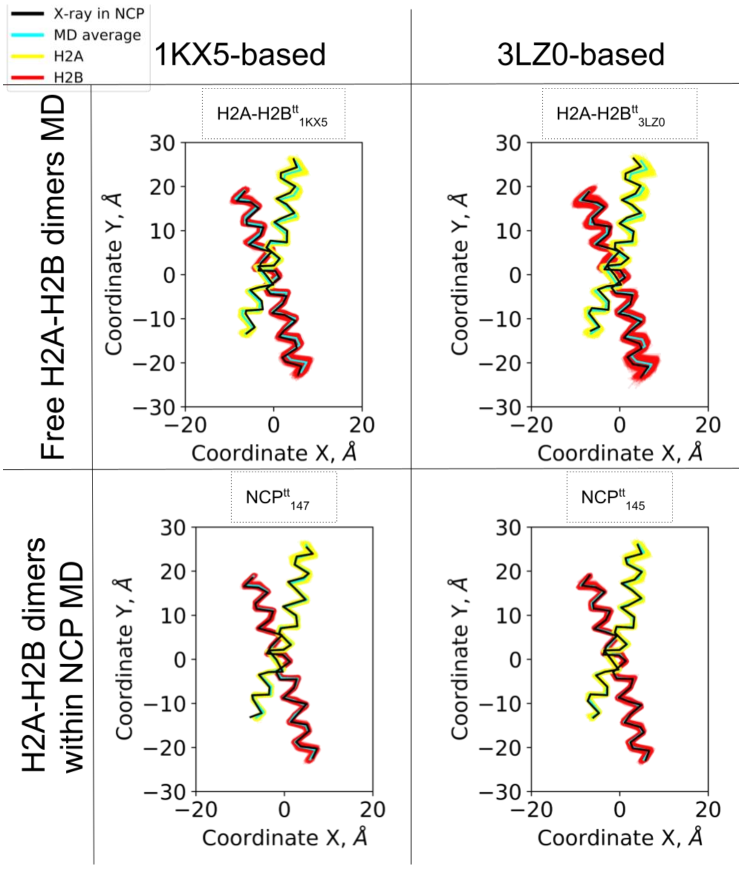

Figure S3. 2D projections of H2A and H2B histones α2-helices' Cα-atoms in the free H2A-H2B dimer and NCP MD simulations.

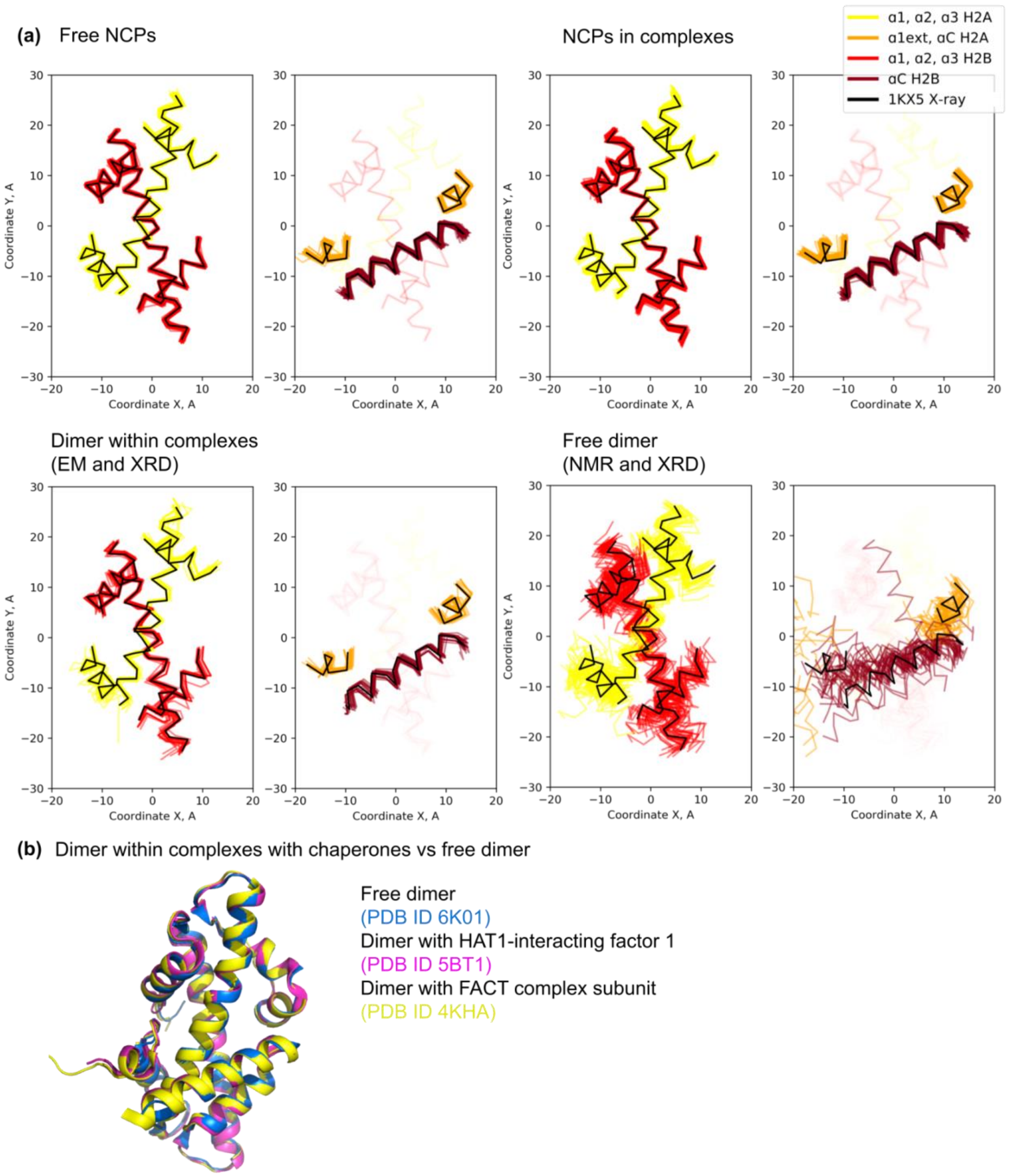

Figure S4. H2A-H2B dimer plasticity in experimental structures from Protein Data Bank. (a) 2D projections of H2A and H2B histone α-helices. The conformations were aligned to the 1KX5-derived H2A-H2B dimer structure positioned in the DRF. **(b)** Overlay of XRD structures of H2A-H2B dimer within complexes with chaperones and free dimer.

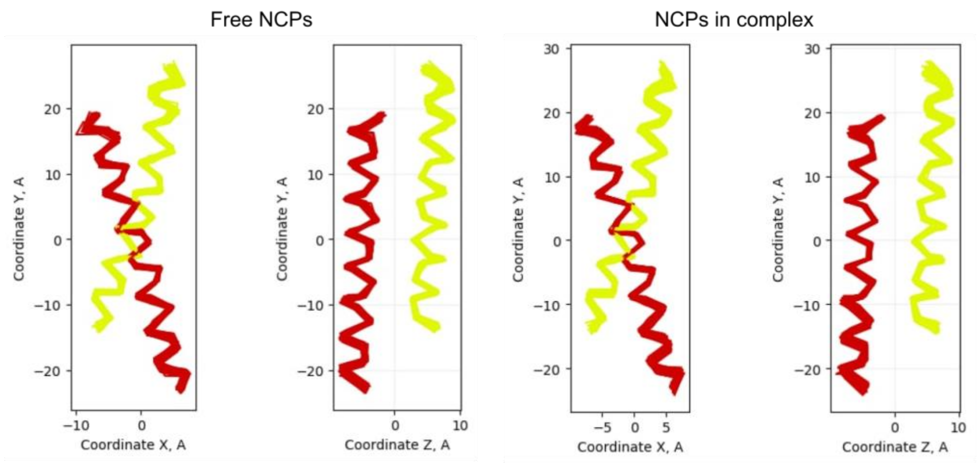

Figure S5. 2D projections of H2A-H2B histones α2-helices' Cα-atoms in experimental structures of NCPs.

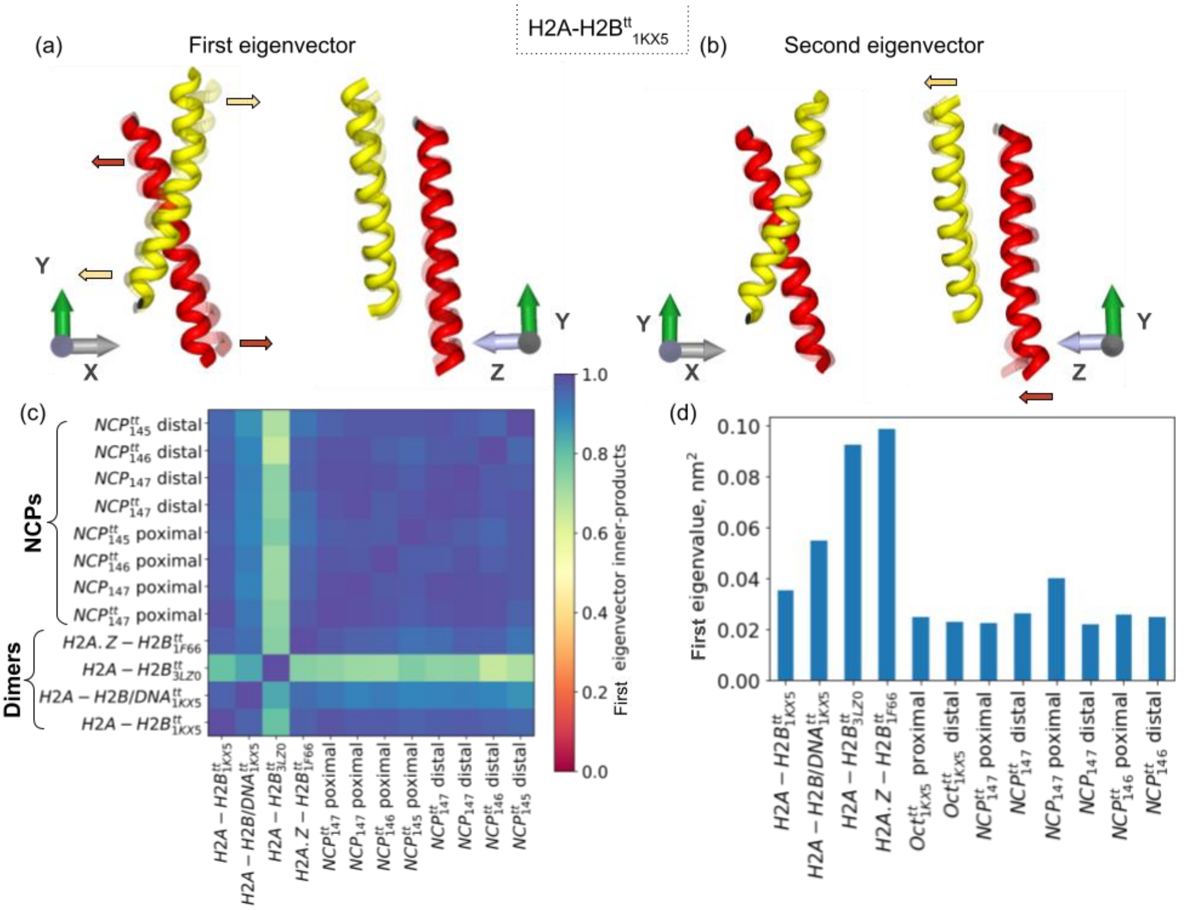

Figure S6. Principal component analysis of H2A and H2B α2-helixes Cα-atoms collective motions of H2A-H2B dimer. (a-b) Representation of first and second eigenvectors of H2A-H2B$^{tt}_{1KX5}$ MD trajectory in two planes (according to DRF). **(c)** Pair-vise inner-products of the first eigenvectors for a set of the free H2A-H2B dimer and NCP-embedded dimer MD trajectories. Vectors were calculated independently using the same method; the first eigenvectors of individual NCP dimers are in the interactive materials. **(d)** Eigenvalues (amplitudes) that correspond to the first eigenvectors of the set of MD trajectories.

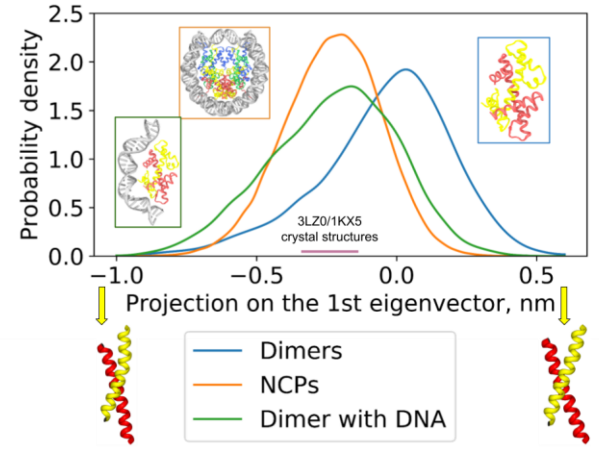

Figure S7. Distribution of MD trajectories' projections on the dimer bending vector (constructed on the first eigenvector of H2A-H2B$_{1KX5}$ dimer MD; extreme conformations of helices are represented under the X-axis).

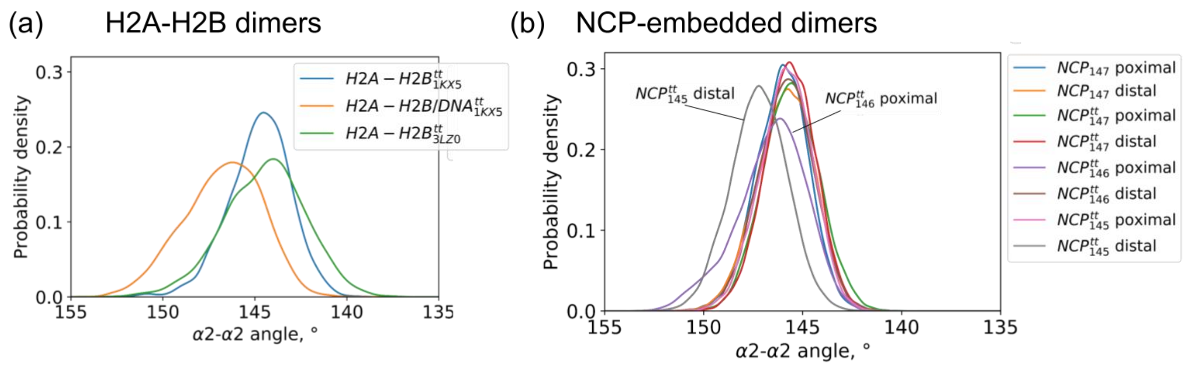

Figure S8. α2-α2 angle values distributions in MD simulations for each independent H2A-H2B dimer.

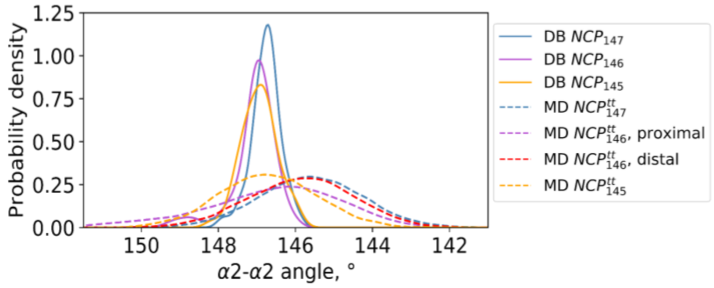

Figure S9. α2- α2 angle values distributions of NCP-embedded H2A-H2B dimers from MD simulations and experimental structures from PDB grouped by DNA sequence (145, 146 or 147 base pairs).

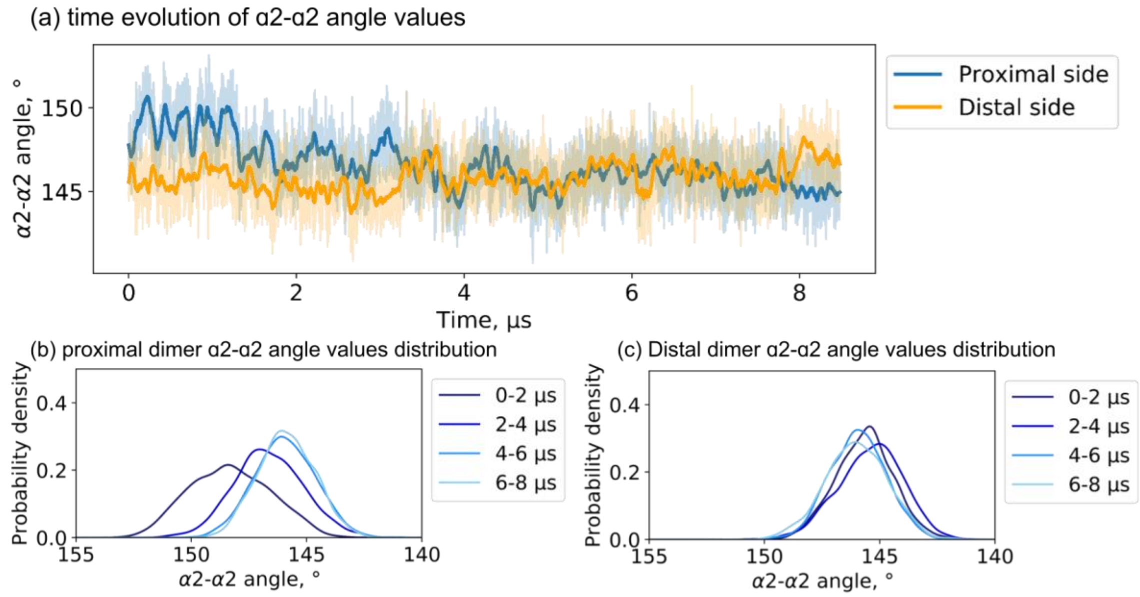

Figure S10. α2- α2 angle values changes in NCP^{tt}_{146} (1AOI-based model) MD simulation.

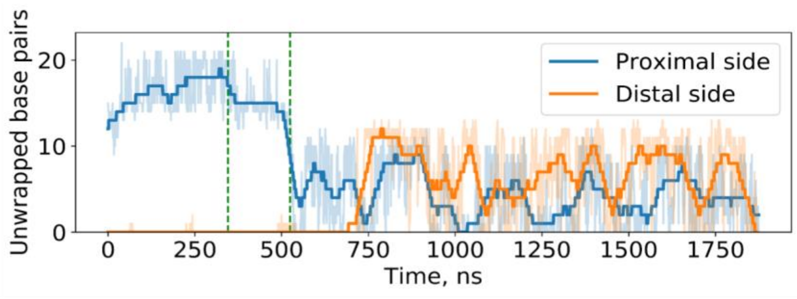

Figure S11. Extent of the DNA unwrapping in MD simulation of NCP with initially unwrapped 25 base pairs DNA segment on the proximal side ($NCP^{tt}_{25\ unwrapped}$). Green vertical lines mark time points of rewrapping events described in Figure 4 d. Thin semitransparent lines are used to plot instantaneous unwrapping values; thick lines depict a smoothed signal with Savitzky-Golay filter using 100 ns window and first-degree polynomial.

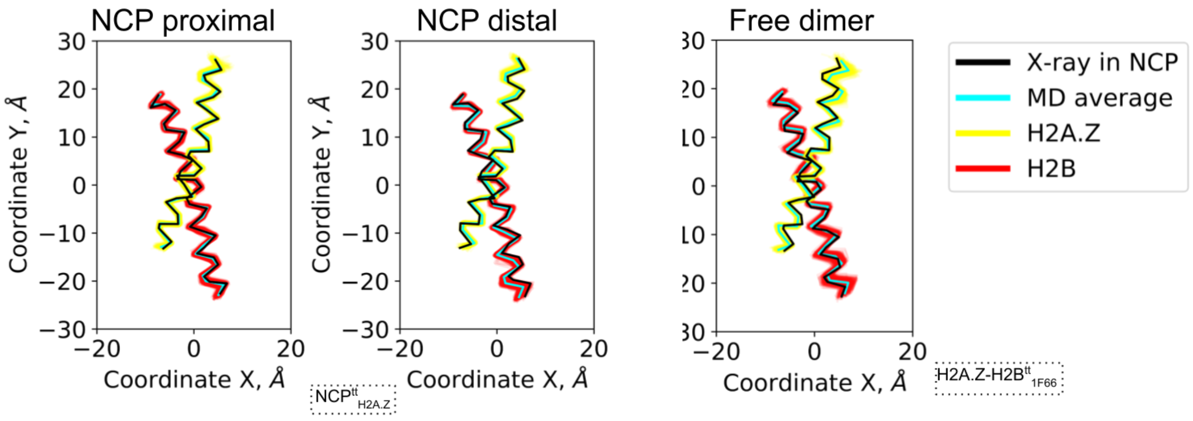

Figure S12. 2D projections of H2A.Z and H2B α2-helices in H2A.Z-H2B dimer and $NCP^{tt}_{H2A.Z}$ simulations.

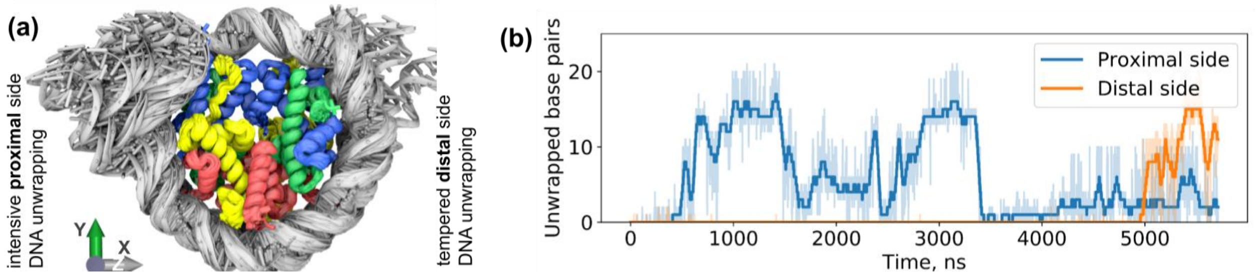

Figure S13. Overview of NCP$^{tt}_{H2A.Z}$ MD simulation. (a) Overlap of MD trajectory frames spaced by 10 ns. (b) DNA unwrapping profile. Thin semitransparent lines are used to plot instantaneous unwrapping values; thick lines depict a smoothed signal with Savitzky-Golay filter using 100 ns window and first-degree polynomial.

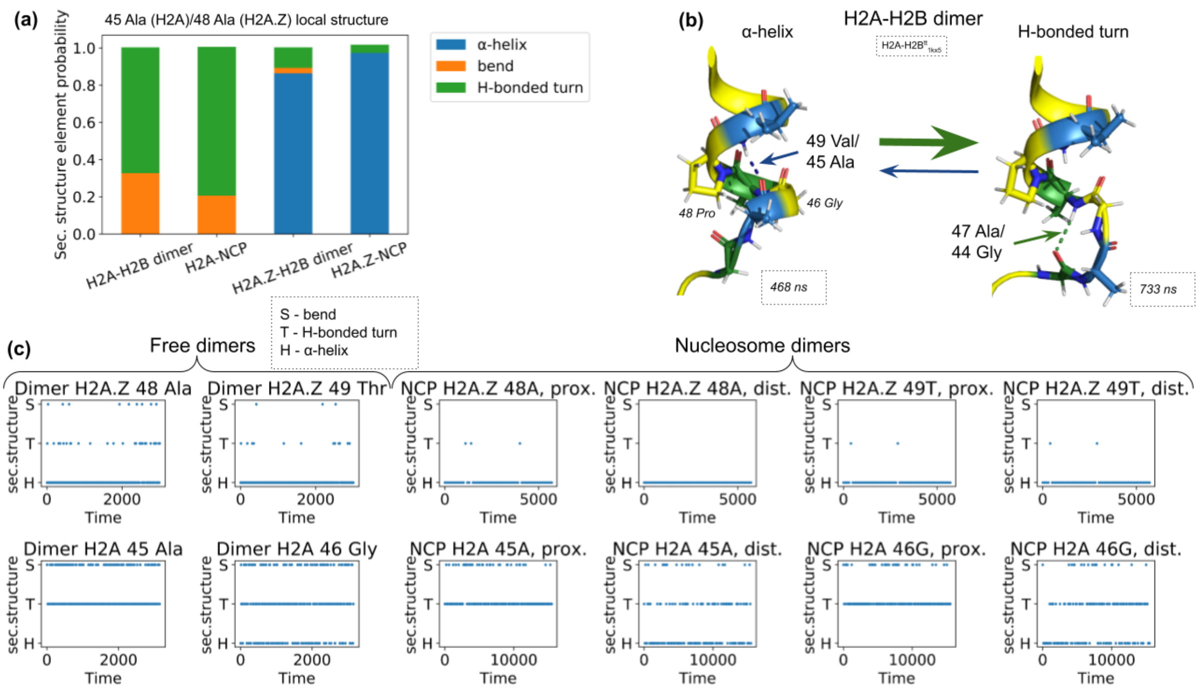

Figure S14. Local secondary structure of α2-helix N-end of H2A and H2A.Z histones. (a) α2-helix N-end local secondary structure elements probability in canonical and variant free dimer and NCP MD simulations. Calculated for 48th residue of the H2A/H2A.Z sequence alignment (Figure 1 d). Secondary structure elements were determined using the DSSP program [2]. **(b)** Zoom-up view of H2A α2-helix N-end in α-helical and turn conformations. Arrows reflect the prevalence of H-bonded turn local structure in H2A-H2B free dimer and NCP-embedded dimers MD. The pairs of residues and their hydrogen bonds specific for each secondary structure element are colored in blue for α-helix and green for H-bonded turn. **(c)** Time evolution of local secondary structure elements in canonical and variant free dimers and NCPs MD.

1. Armeev, G.A.; Kniazeva, A.S.; Komarova, G.A.; Kirpichnikov, M.P.; Shaytan, A.K. Histone Dynamics Mediate DNA Unwrapping and Sliding in Nucleosomes. *Nat. Commun.* **2021**, *12*, doi:10.1038/s41467-021-22636-9.
2. Kabsch, W.; Sander, C. Dictionary of Protein Secondary Structure: Pattern Recognition of Hydrogen-Bonded and Geometrical Features. *Biopolymers* **1983**, *22*, 2577–2637, doi:10.1002/bip.360221211.
